# Draft Genome Sequences of *Tsukamurella* sp. 8F and 8J Strains Isolated from Social Wasps (Vespidae; Polistinae: Epiponini)

**DOI:** 10.1128/mra.00237-23

**Published:** 2023-05-23

**Authors:** Dorian Rojas-Villalta, Kattia Núñez-Montero, Javier Pizarro-Cerdá, Laura Chavarría-Pizarro

**Affiliations:** a Centro de Investigación en Biotecnología, Instituto Tecnológico de Costa Rica, Cartago, Costa Rica; b Facultad Ciencias de la Salud, Instituto de Ciencias Biomédicas, Universidad Autónoma de Chile, Temuco, Chile; c Institut Pasteur, Université Paris Cité, CNRS UMR6047, Yersinia Research Unit, Paris, France; Indiana University, Bloomington

## Abstract

Strains of the genus *Tsukamurella* were isolated from *Polybia* sp. social wasps from Costa Rica. Draft genome sequences from both isolates were obtained of ~4.5 Mb in length and with 68% GC content.

## ANNOUNCEMENT

Bioprospecting for natural products in microorganisms isolated from underexplored environments is a key method to discover new molecules (1). Within these sources, bacterial strains from social insects are promising leads, because these insects have evolved defensive strategies either to prevent pathogen growth or to promote the establishment of symbiotic relationships with microorganisms (2, 3). This suggests the idea of a wide diversity of untapped bioactive metabolites on those symbiotic microorganisms (4). In this context, we studied symbiotic strains of the social *Polybia* sp. wasp, leading to the isolation of strains 8F and 8J from the *Tsukamurella* genus.

Bacterial strains were isolated from the cuticle of *Polybia* sp. wasps collected from Refugio Nacional de Fauna Silvestre Golfito (Costa Rica, 8°39′15.8″N, 83°10′45.1″W) on 30 January 2020. One dead worker wasp was placed onto a petri dish using sterile forceps. Three media were used for isolation of actinobacteria: actinobacterium isolation agar for microbiology (Sigma-Aldrich), International Streptomyces Project 1 (ISP1), and ISP2, all supplemented with nystatin (0.1%) and nalidixic acid (10 μg/mL). These were incubated for 1 month at room temperature (~22°C), and colonies with actinobacterial macroscopic morphologies were isolated on new plates with the same media. Genomic DNA from strains 8F and 8J was extracted by the phenol-chloroform method as described in the work of Chun and Goodfellow (5) from 1 mg of bacterial colonies, aerial mycelia, and spores after 7 days of culture at room temperature. Genome libraries were prepared with the Nextera XT DNA library preparation kit (Illumina) and sequenced twice through a 2- by 150-nucleotide paired-end strategy on the Illumina NextSeq500 platform (Illumina, San Diego, CA, USA). Respective paired raw reads were concatenated to form a single data file for each end. Raw data quality was controlled with FastQC v0.11.9 (6), and reads were trimmed with fastp v0.20.0 (7). Trimmed reads were input for *de novo* assembly using Unicycler v0.4.8 (8). Assembly quality was measured using QUAST v5.0.2 (9) and CheckM v1.1.3 (10). Data were processed with default parameters for all programs used. Additionally, the genomes were annotated with the National Center for Biotechnology Information (NCBI) Prokaryotic Genome Annotation Pipeline v6.5 (PGAP).

Both draft genomes presented a length of ~4.5 Mb. Some relevant genomic characteristics are presented in Table 1. Average nucleotide identity (ANI) comparison was carried out by FastANI v1.32 (11) for available representative genomes for the *Tsukamurella* genus in the National Center for Biotechnology Information (NCBI; accessed 25 January 2023) and our strains (total = 11 genomes). ANI comparison showed a 99.98% similarity within the 8F and 8J strains. However, no other results were above the threshold for defining species (>95%; highest value was Tsukamurella tyrosinosolvens with 79.4% for both our strains). These results suggest that *Tsukamurella* sp. 8F and 8J might be the same new species.

**TABLE 1:** General results of sequencing and genome characteristics of *Tsukamurella* sp. 8F and 8J strains associated with social wasps

Result or characteristic	Strain ID^*a*^	
	*Tsukamurella* sp. 8F	*Tsukamurella* sp. 8J
Total no. of reads (×2)	5,342,838	2,793,831
Total no. of base pairs	801,425,700	419,074,650
Genome size (bp)	4,679,450	4,597,163
No. of contigs	66	86
% GC	68.89	68.88
*N*_50_	138,991	108,455
Completeness (%)	98.31	95.22
Contamination (%)	3.06	2.84
Sequencing depth (×)	171	91
No. of genes annotated with PGAP (12)	4,504	4,444
No. of RNAs annotated with PGAP (12)	1, 1 (16S, 23S)	1, 1 (16S, 23S)
BioSample accession no.	SAMN33554931	SAMN33554932
GenBank WGS accession no.	JARFTP000000000.1	JARFTO000000000.1
GenBank assembly accession no.	ASM2916737v1	ASM2916740v1
Raw read SRA accession no.	SRX19606220	SRX19606221

a Based on ANI results with available representative genomes for the *Tsukamurella* genus in NCBI (accessed 25 January 2023). The species used were *T. asaccharolytica* (ASM785843v1), *T. conjunctivitidis* (ASM785847v1), *T. ocularis* (ASM2480761v1), *T. paurometabola* (ASM9222v1), *T. pseudospumae* (ASM157519v1), *T. pulmonis* (52700 E01), *T. spumae* (ASM1239601v1), *T. sputi* (ASM785844v1), and *T. tyrosinosolvens* (ASM1691946v1). ID, identifier; WGS, whole-genome sequence.

### Data availability.

The whole-genome BioProject PRJNA764377, BioSample material, assembled genomes, and raw reads have been deposited in DDBJ/ENA/GenBank under the accession numbers listed in Table 1.

## References

[1] Matos De Opitz CL, Sass P. 2020. Tackling antimicrobial resistance by exploring new mechanisms of antibiotic action. Future Microbiol 15:703–708. doi:10.2217/fmb-2020-0048. 32648783

[2] Halim M, Aman-Zuki A, Syed Ahmad SZ, Mohammad Din AM, Abdul Rahim A, Mohd Masri MM, Badrul BM, Yaakop S. 2018. Exploring the abundance and DNA barcode information of eight parasitoid wasps species (Hymenoptera), the natural enemies of the important pest of oil palm, bagworm, Metisa plana (Lepidoptera: Psychidae) toward the biocontrol approach and it’s application in Malaysia. J Asia Pac Entomol 21:1359–1365. doi:10.1016/j.aspen.2018.10.012.

[3] Kaltenpoth M, Engl T. 2014. Defensive microbial symbionts in Hymenoptera. Funct Ecol 28:315–327. doi:10.1111/1365-2435.12089.

[4] Beemelmanns C, Guo H, Rischer M, Poulsen M. 2016. Natural products from microbes associated with insects. Beilstein J Org Chem 12:314–327. doi:10.3762/bjoc.12.34. 26977191 PMC4778507

[5] Chun J, Goodfellow M. 1995. A phylogenetic analysis of the genus Nocardia with 16S rRNA gene sequences. Int J Syst Bacteriol 45:240–245. doi:10.1099/00207713-45-2-240. 7537058

[6] Andrews S. 2010. FastQC: a quality control tool for high throughput sequence data. Babraham Bioinformatics. http://www.bioinformatics.babraham.ac.uk/projects/.

[7] Chen S, Zhou Y, Chen Y, Gu J. 2018. fastp: an ultra-fast all-in-one FASTQ preprocessor. Bioinformatics 34:i884–i890. doi:10.1093/bioinformatics/bty560. 30423086 PMC6129281

[8] Wick RR, Judd LM, Gorrie CL, Holt KE. 2017. Unicycler: resolving bacterial genome assemblies from short and long sequencing reads. PLoS Comput Biol 13:e1005595. doi:10.1371/journal.pcbi.1005595. 28594827 PMC5481147

[9] Mikheenko A, Prjibelski A, Saveliev V, Antipov D, Gurevich A. 2018. Versatile genome assembly evaluation with QUAST-LG. Bioinformatics 34:i142–i150. doi:10.1093/bioinformatics/bty266. 29949969 PMC6022658

[10] Parks DH, Imelfort M, Skennerton CT, Hugenholtz P, Tyson GW. 2015. CheckM: assessing the quality of microbial genomes recovered from isolates, single cells, and metagenomes. Genome Res 25:1043–1055. doi:10.1101/gr.186072.114. 25977477 PMC4484387

[11] Jain C, Rodriguez-R LM, Phillippy AM, Konstantinidis KT, Aluru S. 2018. High throughput ANI analysis of 90K prokaryotic genomes reveals clear species boundaries. Nat Commun 9:5114. doi:10.1038/s41467-018-07641-9. 30504855 PMC6269478

[12] Tatusova T, Dicuccio M, Badretdin A, Chetvernin V, Nawrocki EP, Zaslavsky L, Lomsadze A, Pruitt KD, Borodovsky M, Ostell J. 2016. NCBI prokaryotic genome annotation pipeline. Nucleic Acids Res 44:6614–6624. doi:10.1093/nar/gkw569. 27342282 PMC5001611

